# The Field of Science

**Published:** 1894-07-21

**Authors:** 


### The Field of Science.

The foundation-stone of a new hospital at the City Poor House, Aberdeen, was recently laid. The building is to accommodate thirty inmates. The cost of the building, including furnishing, is quoted at £2,500.

---

As affording an example of the practical work which M. Haffkine has effected in India during his tour of anti-choleraic inoculation, we are told that at one village which was visited by the complaint all those who had been treated by M. Haffkine escaped. At this village 116 persons had been inoculated previous to the outbreak. We have already mentioned that a committee of the Calcutta municipality have recommended a sum of money to be voted for two years to aid the further and efficient development of M. Haffkine's scheme.

---

Sir Joseph Lister, Bart., F.R.S., has been awarded the Albert Medal of the Society of Arts, the Prince of Wales giving his approval and sanction to the presentation. The eminent scientist owes this latest distinction to the discovery and establishment of the antiseptic method of treating wounds and injuries, by which, not only has the art of surgery been greatly promoted and human life saved in all parts of the world, but extensive industries have been created for the supply of materials required for carrying the treatment into effect.

---

Our attention has just been drawn by Mr. C. H. Bond to the anatomy of a Chinaman's brain. To its "external" configuration that is. Significant differences exist here, when we compare it with the formation of the average European brain. For instance, the furrows running transversely show a very decidedly more marked prominence than the furrows which run in the antero-posterior direction. The brains upon which Mr. Bond based his investigations were up to the normal standard in convolutional complexity, the frontal lobes being developed, indeed, rather beyond the average. One brain thus studied weighed 1,182 grammes; that is, 176 grammes less than the weight of an average male adult brain. Another very significant point in this field of research is the important and marked difference of the size and weight shown in the cerebrum, as compared to the cerebellum, on which fact Mr. Bond's article on the subject lays much stress.

---

No less than five Italian universities are to be closed —that is to say, abolished, the Government having decided that Italy can in the future dispense with their services, since six of them—Catania, Messina. Modina, Parma, Sienna, and Sarsari only ministered to the intellectual wants of about one hundred students each. The revenues of these former centres of education will be apportioned to the remaining universities in the kingdom, of which more than a sufficient number yet exist.

---

American dentistry does not alone now onfine itself to the preservation or the renewal of teeth, but has extended its operations from the practical into the sphere of the ornamental. Diamonds, we learn, have already been introduced into the front teeth of certain "well-to-do" American citizens. At first hearing, one's sense of the fitness of things is somewhat disturbed by this novelty, but then, after all, what about ear-rings? They have long been accepted in the most civilized countries, and their presence is deemed almost essential for all state occasions; and further, away in less civilized parts of the globe there is the nose ring. So perhaps these particular American dentists are only carrying fashionable female ornamentation to its legitimate ends.

---

An elaborate piece of work is reported of a Parisian dentist. This consisted in stopping the tooth of no less august a personality than that of a performing elephant. The poor beast had suffered considerable pain and discomfort, until the veterinary surgeon in attendance requistioned the services of an efficient dentist. Professor Valadon, who performed the operation—for such it really was—proceeded to drill the cavity to the depth of three inches! For this purpose, of course, the largest and most powerful instruments had to be applied, such as had never previously assisted in an undertaking of such proportions. However, when drilled, the hole was filled with a composition, cased in by a cylinder. Had gold been chosen for this medium, no less than £60 worth would have been required, and the composition which was used has answered all purposes admirably. The elephant, we learn, is now restored to his normal condition.

